# The timing and development of infections in a fish–cestode host–parasite system

**DOI:** 10.1017/S0031182022000567

**Published:** 2022-08

**Authors:** Anika M. Wohlleben, Natalie C. Steinel, Néva P. Meyer, John A. Baker, Susan A. Foster

**Affiliations:** 1 Biology Department, Clark University, Worcester, Massachusetts, USA; 2 Department of Biological Sciences, University of Massachusetts – Lowell, Lowell, Massachusetts, USA

**Keywords:** Alaska, cestode, *Gasterosteus aculeatus*, infection timing, parasite–host interaction, *Schistocephalus solidus*, threespine stickleback

## Abstract

The cestode *Schistocephalus solidus* is a common parasite in freshwater threespine stickleback populations, imposing strong fitness costs on their hosts. Given this, it is surprising how little is known about the timing and development of infections in natural stickleback populations. Previous work showed that young-of-year stickleback can get infected shortly after hatching. We extended this observation by comparing infection prevalence of young-of-year stickleback from 3 Alaskan populations (Walby, Cornelius and Wolf lakes) over 2 successive cohorts (2018/19 and 2019/20). We observed strong variation between sampling years (2018 *vs* 2019 *vs* 2020), stickleback age groups (young-of-year *vs* 1-year-old) and sampling populations.

## Graphical abstract

**Figure:**
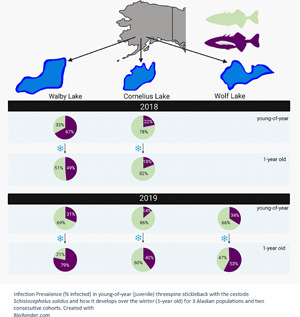

## Introduction

Many parasites have complex life cycles, infecting several hosts before reaching sexual maturity. Infection success is dependent on several factors, including timing of transmission and environmental conditions (Wolinska and King, 2009; Karvonen *et al*., 2013; Stutz *et al*., 2015; Franke *et al*., 2017). Further, population-level differences in host resistance contribute to differential infection prevalence (Hamley *et al*., 2017; Weber *et al*., 2017*a*; Piecyk *et al*., 2021). In the current study, we used a fish–cestode host–parasite system to investigate when infection occurs and to examine changes in infection prevalence (% of infected fish) during the winter when the host has fewer resources.

### The study system

Following the last glacial maximum, oceanic threespine stickleback (*Gasterosteus aculeatus*) colonized freshwater habitats where they had to adapt to new environmental conditions, including new parasites. The cestode *Schistocephalus solidus* is trophically transmitted with a 3-host life cycle. The free-swimming, first larval stage (coracidium) hatches in freshwater and, after ingestion by the first intermediate host, a cyclopoid copepod develops into the second larval stage (procercoid). After the second intermediate host, the threespine stickleback, consumes the infected copepod, *S. solidus* will penetrate the fish's gut wall and develop into the third larval stage (plerocercoid). Most of the parasite's growth occurs in the stickleback host and the threshold weight for successful reproduction is around 50 mg (Tierney and Crompton, 1992). When the infected stickleback is ingested by the final host, a warm-blooded vertebrate and typically a piscivorous bird, reproduction occurs rapidly (Tierney and Crompton, 1992; Barber *et al*., 2008). The tapeworm's eggs are released into the water with the bird's feces within a few days of infection and the cycle begins again. Alaskan stickleback typically spawn from mid-May through June (Heins *et al*., 1999), and females produce several clutches per breeding season. Shortly after hatching, young-of-year stickleback (juveniles that are <1 year old) start eating macroinvertebrates, and stickleback as small as 15 mm feed heavily on copepods, the potential host of *S. solidus*. As the stickleback grow, they consume fewer copepods (Milinski and Christen, 2005), making it likely that stickleback become infected early during their lives (Pennycuick, 1971; Christen and Milinski, 2003; Heins *et al*., 2016). Heins *et al*. (2016) provided empirical evidence that young-of-year stickleback in Cheney Lake (AK, USA) were infected during their first summer. Pennycuick (1971) found a similar pattern in Priddy Pool (Somerset, England), where over 78% of young-of-year stickleback were infected within months after hatching. Both of these studies only tracked individual populations, but we know that: (1) different freshwater populations of stickleback exhibit very different *S. solidus* loads (Pennycuick, 1971; Heins and Baker, 2003, 2011, 2014; Heins *et al*., 2010, 2011; Morozińska-Gogol, 2011; Weber *et al*., 2017*a*), and (2) there can be strong variation in infection intensity within lakes between years (Pennycuick, 1971; Heins and Baker, 2011).

The mechanisms behind the population-level variation in *S. solidus* infections are an area of ongoing research. It was long assumed that the high mobility of the definitive bird host would lead to high gene flow/migration rates in *S. solidus* and consequently results in a panmictic genetic structure (reviewed in Nadler, 1995). However, recent work (Sprehn *et al*., 2015) found evidence for fine-scale population structure of *S. solidus* in the Cook Inlet region of southcentral Alaska. It is assumed that these observed patterns of genetic variation in *S. solidus* may represent adaptions to host genotypes. Indeed, evidence suggests co-evolution between hosts and parasites on different geographical scales (Hamley *et al*., 2017; Weber *et al*., 2017*a*; Piecyk *et al*., 2021). Additionally, infections measured in the laboratory and in the wild have shown that the environment in which hosts and parasites interact substantially affects the strength and specificity of selection, and different components of host–parasite fitness seem to be differentially altered by the environment (Wolinska and King, 2009; Karvonen *et al*., 2013; Stutz *et al*., 2015; Franke *et al*., 2017). To further our knowledge regarding changes in natural infection levels over time and importantly across different populations, we studied 3 different Alaskan stickleback populations for 2 consecutive cohorts: 2018/19 and 2019/20, measuring the infection rate in young-of-year and 1-year-old stickleback. We chose stickleback populations with different adult infection rates (Heins *et al*., 2010, unpublished data) and were interested if these differences were already detectable in young-of-year fish. We expected that young-of-year fish from the high infection population would already harbour more *S. solidus* larvae than young-of-year fish from the low infection population. Further, we hypothesized that infection prevalence would decrease over the winter as infected sticklebacks should have an increased mortality compared to uninfected sticklebacks.

## Methods

Collections and procedures were approved by annual Aquatic Resource Permits from the Alaskan Department of Fish and Game (P-18-011, P-19-0111, P-20-004), and IACUC approved animal care protocols (013R).

We chose Walby, Cornelius and Wolf lakes in southcentral Alaska for this study as they are easily accessible for repeated sampling and adult fish in Walby Lake and Cornelius Lake show strong differences in their *S. solidus* infection rates. Over a period of 7 years (1996, 1999–2004), the mean infection prevalence (% infected fish) in Cornelius fish was 7 ± 4% (standard deviation) (unpublished data). The mean infection prevalence in Walby fish was 50 ± 20% (standard deviation) over a sampling period of 8 years (1996–2003) (Heins *et al*., 2010). No data were available on infection prevalence in Wolf fish. Since the study already included 1 high and 1 low infection lake, we decided to include a lake without prior knowledge of the infection intensity. To assess infections of the 2018 cohort, we sampled Walby Lake and Cornelius Lake in August 2018 and June 2019. To assess infections of the 2019 cohort, we sampled all 3 lakes in August 2019 and June 2020. The lake coordinates and sample sizes are listed in Table 1.

**Table 1.** Lake coordinates and sample sizes for young-of-year (yoy) and 1-year-old (1 yo) stickleback from cohort 2018 (yoy-2018 and 1 yo-2019) and cohort 2019 (yoy-2019 and 1 yo-2020) for the sample lakes

Lake	Coordinates	yoy – 2018	1 yo – 2019	yoy – 2019	1 yo – 2020
Cornelius	61°37′50.16″N, 149°15′10.08″W	101	71	107	149
Walby	61°37′9″N, 149°12′39″W	106	123	106	149
Wolf	61°38′42″N, 149°16′40.08″W	NA	NA	106	137

### Young-of-year stickleback infection rate

In mid-August of 2018 and 2019, we caught young-of-year stickleback (juveniles that are <1 year old) based on size, using 4-inch aquarium nets in shallow areas of the sampled lakes. In August, young-of-year stickleback should be between 2 and 3 months old, depending on the time of hatching during the spawning season (Heins *et al*., 1999). On the day of collection, we euthanized the stickleback with an overdose of MS-222 (Tricaine-S, Syndel, Ferndale, USA), and determined the infection status based on a protocol previously established by Wohlleben *et al*. (2018). In short, following dissection, we screened the body cavity and organs for *S. solidus* under a dissection microscope [Trinocular Zoom Stereo Microscope, EMZ-TR (0.7×–4.5×), Meiji, Tokyo, Japan]. If no *S. solidus* were discovered, we rinsed the body cavity with water into a Petri dish and screened the dish and the body cavity immediately. If we still could not find any parasites, we incubated the dissected stickleback in water at room temperature for about 30 min, and subsequently screened again. Each stickleback was either screened 3 times by 2 different people or until *S. solidus* parasites were found. This method was found to be reliable for detecting parasites as early as 24 h after infection (Wohlleben *et al*., 2018).

### One-year-old stickleback infection rate

We collected stickleback 1 year and older from the sampled lakes in the first 2 weeks of June 2019 and 2020 using unbaited, 6 mm wire-mesh minnow traps set for a day at different locations near shore. We euthanized the fish until quiescent with MS-222 and stored them in 10% buffered formalin until further examination. At Clark University, we dissected the fish and examined the body cavities for any plerocercoids under a dissection microscope [Trinocular Zoom Stereo Microscope, EMZ-TR (0.7×–4.5×), Meiji]. We removed, counted and, if possible, weighed (to the neatest 0.1 mg) all parasites (Table S1) with a digital scale (College Precision Balance, B303-S, Mettler Toledo, Greifensee, Switzerland). We used a dial calliper (Dial Calliper, 505-647, Mitutoyo, Kawasaki, Japan) to measure the standard length (to the nearest 0.1 mm; body length from the tip of the nose to the last vertebrae) and recorded the weight before and after dissection (to the nearest 0.1 mg) (College Precision Balance, B303-S, Mettler Toledo) of all stickleback.

### Calculating age distribution of 1-year and older stickleback

In spring, several age groups of stickleback are present in the same lake, and random sampling results in the capture of mixed age groups. Therefore, we had to determine the age distribution in our samples to assess the infection rate of 1-year-old fish. Typically, when determining age-based growth for fish, weight is preferred over length since the latter slows down throughout life, whereas weight continues to increase nearly linearly (Baker *et al*., 2008). However, *S. solidus* infection is likely to reduce the weight of the host fish, which should lead to a biased weight distribution. Therefore, for this experiment, length was chosen as it should be a more accurate measure (Barber and Svensson, 2003; Heins *et al*., 2011, 2016). To estimate the size that appears to separate 1-year-olds from older stickleback, we grouped the stickleback based on their standard length and initially assessed the distribution visually (Fig. S1). To verify these first estimates, we used a maximum likelihood-based method and grouped the data with a combination of a Newton-type algorithm and the EM algorithm (MacDonald and Pitcher, 1979). We used the population mean plus the population standard deviation as the age determinant for 1-year-old stickleback. With this method we likely excluded some 1-year-old stickleback, but the certainty is higher that no 2-year-old stickleback were incorporated in downstream analyses. The exact cut-off values and statistical data are shown in Table 2. This method of determining age did not work for the samples from Cornelius Lake since relatively few 1-year-old compared to older fish were caught during sampling. Therefore, we used 7 years of dissection data that were originally collected for other purposes (unpublished data) to determine the cut-off size for 1-year-old stickleback as described above.

**Table 2.** Population parameters and maximum cut-off values for 1-year-old stickleback

Lake	Cohort	Age	Population mean (*μ*)	Population standard deviation (*σ*)	Cut-off size (mm)
Cornelius – original	2018	1	45.60	5.945	NA
		2	54.55	7.113	
Cornelius – collection	2018	1	37.86	3.897	41.8
		2	51.03	5.252	
Walby	2018	1	35.57	2.711	38.3
		2	44.05	3.357	
Cornelius	2019	1	38.75	3.087	41.8
		2	51.96	4.140	
Walby	2019	1	41.42	3.427	44.8
		2	50.32	4.164	
Wolf	2019	1	45.65	4.951	50.6
		2	63.86	6.927	

Cornelius Lake samples from cohort 2018 (Cornelius – original) could not be used to determine age cut-off values (lack of small fish in the samples), therefore 7 years of data from Cornelius Lake fish (Cornelius – collection) were used instead. The cut-off value is the population mean + the standard deviation. Population mean and standard deviation were calculated using a maximum likelihood-based approach.

### Statistics

All statistical analyses were performed in the R statistics software (Version 4.0.3) (R Core Team, 2020). All R packages used are listed in the Supplementary Material (Table S2). To test for differences in infection prevalence (dependent variable) between lakes (independent variable) within age group and cohort, we ran a binomial logistic regression using generalized linear models (Crawley, 2012). Since there were only 2 layers of the independent variable (Lake) in 2018, we did not calculate a *post-hoc* comparison. For the comparison of infection prevalence within stickleback age groups for the 2019 cohort, we calculated a Tukey-corrected *post-hoc* pairwise comparison. Similarly, to assess the differences in infection prevalence between stickleback age groups within cohort and lake, we ran binomial logistic regressions with lake and age as independent variables and infection prevalence as the dependent variable. We calculated a Tukey-corrected *post-hoc* pairwise comparison. We analysed differences in mean parasite weight and mean parasite number per fish (Table S1) in the same way as described above.

## Results

### Development of infection over the winter

**Fig. 1.**
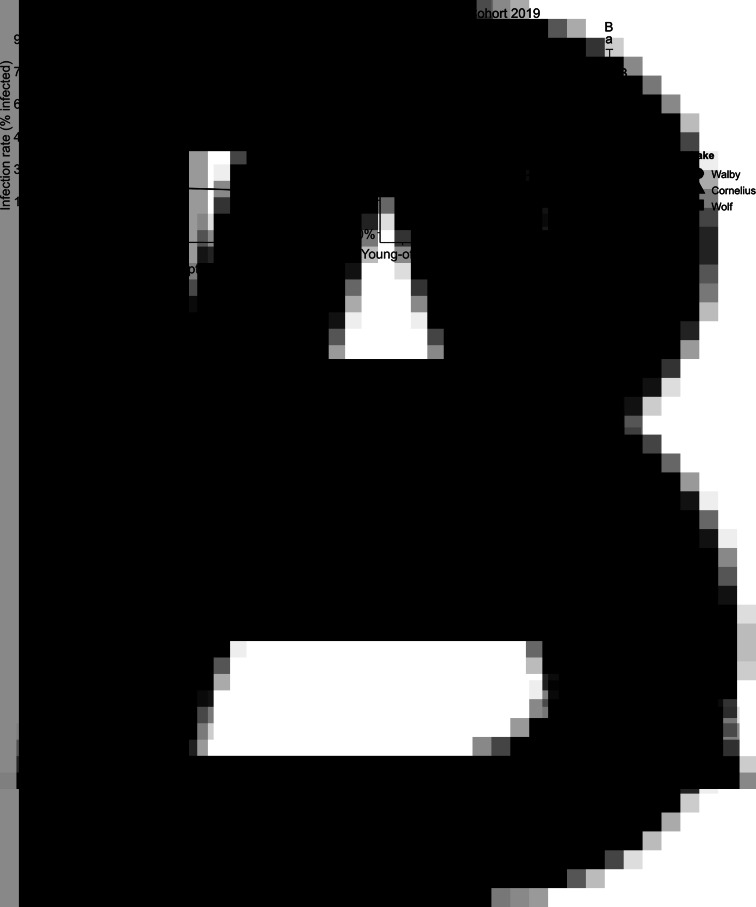
Probability of being infected with *Schistocephalus solidus* (% infected fish) for young-of-year and 1-year-old stickleback. (a) Cohort 2018; (b) cohort 2019. The error bars represent 95% confidence intervals and were calculated using a logit regression. Capital letters above error bars indicate statistically significant differences between age groups within lake and cohort. Lower case letters indicate statistically significant differences between lakes within age group and cohort. Sample size (cohort 2018) yoy Walby, 106; yoy Cornelius, 101; 1 yo Walby, 123; 1 yo Cornelius, 71. Sample size (cohort 2019) yoy Walby, 106; yoy Cornelius, 107; yoy Wolf, 106; 1 yo Walby, 149; 1 yo Cornelius, 149; 1 yo Wolf, 137.

In the 2018 cohort, the infection prevalence between young-of-year (caught in August 2018) and 1-year-old (caught in June 2019) stickleback decreased significantly in the high-infection Walby Lake (yoy: 66.98%, 1 yo: 48.67%), but we found no significant change in the low-infection Cornelius Lake (yoy: 21.78%, 1 yo: 19.3%) (Table 3, Fig. 1). In the 2019 cohort, we observed a significant increase in infection prevalence in Walby Lake (yoy: 31.13%, 1 yo: 78.41%) and Cornelius Lake (yoy: 14.02%, 1 yo: 39.66%) over the winter. Despite a slight increase in infection prevalence in Wolf Lake (yoy: 33.96%, 1 yo: 41.79%), this change was not statistically significant (Table 4, Fig. 1).

**Table 3.** Statistical results for comparisons of cohort 2018 fish using a binomial logit regression

Comparison	Standard error	*z* ratio	*P* value
yoy Walby–yoy Cornelius	0.317	−6.255	<0.0001***
1 yo Walby–1 yo Cornelius	0.356	−4.064	<0.0001***
yoy Cornelius–1 yo Cornelius	0.39	−0.556	0.9449
yoy Walby–1 yo Walby	0.274	−2.757	0.0297*

yoy, young-of-year; 1 yo, 1-year-old.

*P < 0.05, ***P < 0.001

**Table 4.** Statistical results for comparisons for cohort 2019 fish using a binomial logit regression and a Tukey-corrected *post-hoc* comparison

Comparison	Standard error	*z* ratio	*P* value
yoy Walby–yoy Cornelius	0.349	−2.925	0.0181*
yoy Walby–yoy Wolf	0.293	−0.440	0.9716
yoy Cornelius–yoy Wolf	0.346	−3.322	0.005**
1 yo Walby–1 yo Cornelius	0.26	−6.498	<0.0001***
1 yo Walby–1 yo Wolf	0.263	4.431	<0.0001***
1 yo Cornelius–1 yo Wolf	0.239	−2.198	0.0715
yoy Cornelius–1 yo Cornelius	0.325	4.371	0.0002**
yoy Walby–1 yo Walby	0.289	7.221	<0.0001***
yoy Wolf–1 yo Wolf	0.267	2.981	0.0341*

yoy, young-of-year; 1 yo, 1-year-old.

*P < 0.05, **P < 0.01, ***P < 0.001

### Differences in infections between lakes

Comparison of the young-of-year stickleback from the 2018 cohort revealed a significant difference in infection rate between the 2 lakes: young-of-year stickleback from Walby Lake were more likely to be infected with *S. solidus* than stickleback from Cornelius Lake (Walby: 66.98%, Cornelius: 21.78%). One-year-old fish from the 2018 cohort showed the same pattern (Walby: 48.67%, Cornelius: 19.3%) (Table 3, Fig. 1). Comparison of the young-of-year fish from the 2019 cohort (Walby, Cornelius and Wolf lakes) showed a significant association between infection rate and lake (Table 4, Fig. 1). Young-of-year fish from Cornelius Lake had a lower probability of being infected compared to fish from the other 2 sample lakes (Walby: 31.13%, Cornelius: 14.02%, Wolf: 33.96%). In the 1-year-old fish, the infection prevalence of fish from Cornelius Lake and Wolf Lake resembled each other, while fish from Walby Lake had a higher chance of being infected with *S. solidus* (Walby: 78.41%, Cornelius: 39.66%, Wolf: 41.79%) (Table 4, Fig. 1). The mean parasite number and mean parasite weight per fish did not differ for the 1-year-old stickleback between lakes (Table S1).

## Discussion

In the present study, we examined the infection prevalence of young-of-year stickleback with the cestode *S. solidus* and how this changed over winter for the same cohort. Stickleback from 3 Alaskan populations had *S. solidus* infections only few months after hatching, showing strong variation in infection prevalence between sampling years and age groups.

### Infection of young-of-year threespine stickleback and changes over the winter

Within a few months of hatching, young-of-year stickleback already harbour *S. solidus* plerocercoids (Fig. 1). This is not surprising considering small stickleback predominantly eat copepods, the first intermediate host of *S. solidus* (Pennycuick, 1971; Christen and Milinski, 2003; Heins *et al*., 2016). However, this observation raises questions regarding the fitness of these parasites, as they now find themselves in hosts that are too small for the parasite to grow to sexual maturity (>50 mg) (Tierney and Crompton, 1992). The parasite has reached a reproductive dead end unless it can allow its host to grow to an appropriate size (Christen and Milinski, 2003). Even if parasites smaller than 50 mg are capable of maturing in the final host (Heins *et al*., 2002), the small size of young-of-year fish in the sampled lakes may prevent the parasite from reaching a size required for sexual maturity. Similarly, previous research has shown that high infection intensities within a single stickleback host can cause large numbers of *S. solidus* larvae to fail to reach a size that is large enough to mature in the final avian host due to both energy and space constraints (Heins and Baker, 2011). Christen and Milinski (2003) found that infected stickleback still allocate resources towards growth even though *S. solidus* parasites convert energy more efficiently than stickleback (Walkey and Meakins, 1970). This could indicate an adaptive life-history strategy of restrained growth in *S. solidus* – a parasite that grows too quickly or that is too virulent might overexploit its intermediate host and risk damaging or killing it before being transmitted to the next host (Christen and Milinski, 2003).

We observed a significant decrease in infection prevalence in young-of-year Walby fish over the winter in the 2018 cohort, but not in Cornelius fish. However, in the 2019 cohort, the infection prevalence increased significantly over the winter in both Walby and Cornelius fish, with no change in Wolf fish (Fig. 1). The different trends in the same lakes for 2 consecutive years suggest the development of infection prevalence over the winter is not as easily predictable as we hypothesized. It would be useful to examine the parasite load per fish (parasite weight or parasite number), since fish with fewer or smaller parasites should survive the winter better. For our study, no difference in the mean parasite number nor mean parasite weight per fish was found in the 1-year-old stickleback (Table S1). Unfortunately, we did not collect data on the parasite weight and number in young-of-year stickleback. *Schistocephalus solidus* infection intensity and host:parasite weight ratios have been found to vary between years (Heins *et al*., 2010), and we are therefore careful about interpreting this finding.

We observed an increase in infection prevalence over the winter in Walby and Cornelius fish in the 2019 cohort. However, there is no solid evidence of increased survivorship of infected fish over the winter (reviewed in Barber and Scharsack, 2010). In fact, on restricted diets infected stickleback seem to die earlier than non-parasitized fish (reviewed in Barber *et al*., 2008). The increase in infections likely results from young-of-year stickleback feeding on (infected) copepods after our August sampling, leading us to underestimate the actual number of infections that occurred before lakes freeze over, typically between October and December (Woods, 1985). Heins *et al*. (2016) observed that infection rates continued to climb throughout autumn, with around 30% of young-of-year Cheney Lake (AK) fish infected with *S. solidus* in mid-September, and nearly 50% of young-of-year infected by mid-October. In the same study, the infection prevalence nearly doubled between mid-October and the following June. Copepods are unlikely to acquire new infections under ice since the development of fully formed *S. solidus* embryos is highly temperature-dependent, and the hatching of coracidia may not occur at temperatures below 5°C (Dubinina, 1957; Mason, 1966). Once hatched, the free-living coracidium needs to be ingested by the first host within days (Thomas, 1947; Dubinina, 1957; Smyth and Clegg, 1959). The only possibility for stickleback to become infected under the ice cover is from a reservoir of infected copepods. Cyclopoid copepods lower their metabolism at low temperatures (Maier, 1990), and unfortunately the interaction between *S. solidus* and its first intermediate host is extremely understudied, so it is unclear how long the copepod host can stay infected and successfully transmit *S. solidus*. We are aware that early after infection, *S. solidus* larvae are still relatively small (~100 *μ*m), and detection is difficult but can be accurate if the parasites are still alive (Wohlleben *et al*., 2018). For this reason, the infection rate was assessed right after euthanizing young-of-year stickleback. During the dissection, only parasites in the body cavity but not the stomach were counted.

### Differences in infections between lakes

Due to the independent colonization of freshwater habitats by marine stickleback, resistance to *S. solidus* infections varies among lakes and host populations (e.g. Weber *et al*., 2017*a*, 2017*b*). Consistent with previous observations in adult Walby and Cornelius fish, young-of-year stickleback from Walby Lake (high infection population) already showed a higher infection prevalence than fish from Cornelius Lake (low infection population) (Fig. 1). As there were no previous reports on infection prevalence or intensity on Wolf fish, we had no prior expectations. Currently, there is little information about the systemic responses in stickleback originating from the 3 sampling lakes, but an increasing number of studies in different Canadian and European stickleback populations suggests population-specific responses in the innate and adaptive immune system following exposure to *S. solidus* (Lohman *et al*., 2017; Fuess *et al*., 2021), and recent work examines the role of fibrosis-induced suppression of parasite growth in some stickleback populations (Weber *et al*., 2017*a*). Having established that young-of-year stickleback are able to be infected, it is important to focus future research on population-level differences in the immune response of infected and uninfected stickleback during different time points post infection, starting as soon after hatching and parasite exposure as possible.

According to previous research in laboratory-bred stickleback, only fish smaller than 38 mm will ingest copepods (Christen and Milinski, 2003) and consequently lakes with generally smaller stickleback might be more likely to become infected. We however did not find this pattern in the current study, as 1-year-old fish in Walby Lake (cohort 2019) had the second largest average size, after those in Wolf Lake, but had the highest infection prevalence (Fig. 1). All 3 of the sampling lakes are ecologically very similar apart from their depths: The average depth of Walby Lake and Wolf Lake is 1.7 and 2.1 m, respectively, with both reaching a maximal depth of not more than 5.5 m. In contrast, the low-infection Cornelius Lake reaches an average depth of 7 m, with a maximal depth of 16.5 m (Alaska Department of Fish and Game). In deep lakes such as Cornelius Lake, typically a thermocline forms, and *S. solidus* eggs deposited into limnetic areas may sink below the hypolimnion where they would be subject to low temperatures and light conditions. This should at least hamper if not prevent egg development and would result in a reduced infection pressure for stickleback. The infection rate in both Walby and Cornelius lakes was lower in young-of-year fish in 2019 compared to 2018. This is not surprising as there is strong evidence that parasite loads can vary substantially between years (Pennycuick, 1971; Heins *et al*., 2010, 2011; Heins and Baker, 2011; Morozińska-Gogol, 2011; Weber *et al*., 2017*a*). Although the mechanisms behind these fluctuations are not known yet, it has been shown that high infection prevalence can result in a crash in population size of the stickleback host, causing a dramatically reduced transmission of *S. solidus* and hence a reduction of infections in the following years (Heins and Baker, 2011; Heins *et al*., 2010). Due to time and money restrictions, data about population size were not collected, and therefore it is uncertain if the decrease in infection prevalence observed was caused by a dip in stickleback abundance.

### Concluding remarks

In summary, we demonstrate here that young-of-year fish from the high infection population (Walby Lake) already had higher infection rates than fish from the low infection population (Cornelius Lake). By comparing multiple populations, we also showed that there is variability in infection prevalence between the sampled lakes as well as in the direction in which the infection prevalence changes over the winter. It is not clear if (un)infected fish survive disproportionately or if stickleback can acquire new infections over the winter, and future studies on this topic are needed. Tracking individual stickleback over the winter and assessing survival and infection status could be a first step, extending existing work by Heins and Baker (2011). Most importantly, we demonstrate that large numbers of stickleback in 3 Alaskan populations were infected with *S. solidus* only a few months after hatching, supporting previous reports from Pennycuick (1971) and Heins *et al*. (2016). The timing of infection is likely to vary globally with climate and the length of host and parasite life cycles. Future studies should focus on pin-pointing the timing of infection in more stickleback populations in different parts of the northern hemisphere. Further, we propose that the finding that Alaskan stickleback are infected within few months of hatching should be considered in the design of future lab infection studies. To generate data that are more applicable to natural field situations, it seems necessary to pay attention to the natural timing of infection for different wild populations.

## Data Availability

Data available upon request.

## Supplementary material

For supplementary material accompanying this paper visit https://doi.org/10.1017/S0031182022000567. click here to view supplementary material
