# Supplementary material for: The timing and development of infections in a fish–cestode host–parasite system
Source: Parasitology. 2022 May 16;149(9):1173–8. doi: 10.1017/S0031182022000567 (PMC10090619; doi:10.1017/S0031182022000567)
Supplement: Supplementary file 1 [file S0031182022000567sup001.docx]

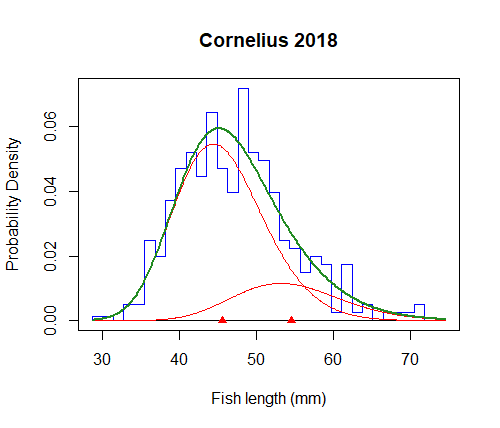

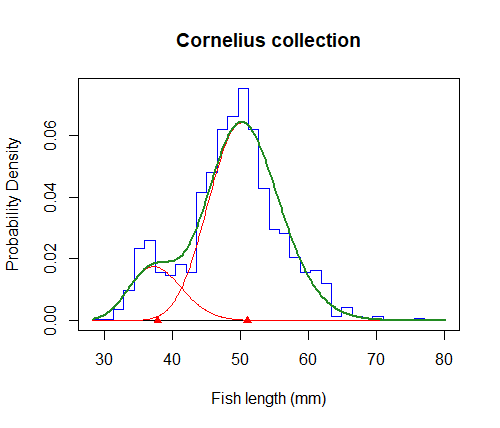


*
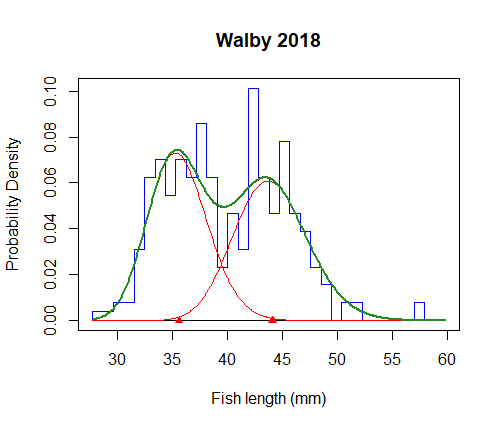
*
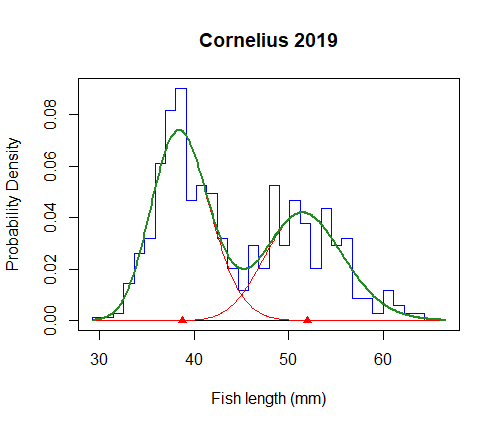


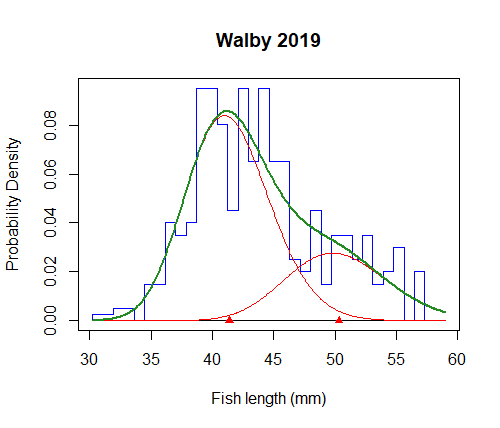

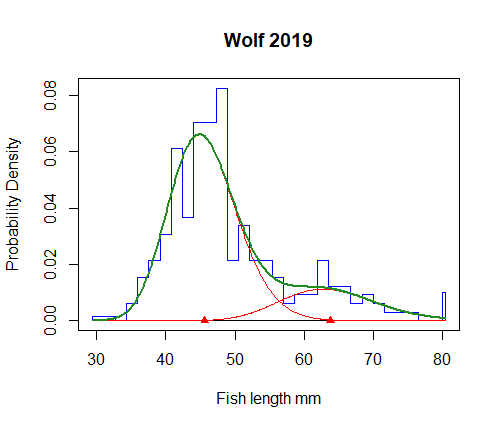


Figure S1: Probability Density plots to assess adult stickleback size distribution for the sample lakes. The blue bars represent the size bins, the green curve represents a smoothed curve of the bins, and the red distributions are the age groups as calculated by the model (mixdist).

Table S1: R-packaged used for statistical analyses and plot creation

| R-package | Used for… |
| --- | --- |
| xlsx (Dragulescu and Arendt, 2020) | Importing excel files |
| mixdist (Macdonald and Du, 2018) | Age class analysis |
| lme4 (Bates et al. 2015) | Linear mixed effects modelling |
| emmeans (Lenth 2021) | Post-hoc comparisons |
| ggplot2 (Wickham 2016) | Plot creation – Data visualization |
| sjPlot (Luedecke 2021) | Plot creation from regression model |
| scales (Wickham and Seidel 2020) | Plot creation – Scales function for visualization |

Table S2: Mean Parasite number and mean parasite weight per fish for1-year-old stickleback from the Cohort 2018 and Cohort 2019. SD = standard deviation

| Lake | Parasite number  Cohort 2018 | | Parasite weight per fish (g) – Cohort 2018 | | Parasite number  Cohort 2019 | | Parasite weight per fish (g) – Cohort 2019 | |
| --- | --- | --- | --- | --- | --- | --- | --- | --- |
|  | Mean | SD | Mean | SD | Mean | SD | Mean | SD |
| Walby | 1.56 | 0.9 | 0.13 | 0.11 | 1.9 | 1.5 | 0.2 | 0.09 |
| Cornelius | 2.18 | 2.79 | 0.12 | 0.11 | 1.44 | 1.14 | 0.1 | 0.09 |
| Wolf | NA | NA | NA | NA | 1.69 | 1.06 | 0.22 | 0.08 |

# References (Supplementary Material)

**Bates D, Maechler M, Bolker B and Walker S** (2015) Fitting Linear Mixed-Effects Models using lme4. *Journal of Statistical Software*, **6**, 1-48. doi:10.18637/jss.v067.i01

**Dragulescu A and Arendt C** (2020) xlsx: Read, Write, Format Excel 2007 and Excel 97/2000/XP/2003 Files. Rpackage version 0.6.5. Available at https://CRAN.R-project.org/package=xlsx

**Length RV** (2021) emmeans: Estimated Marginal Means, aka Least-Squares Means. Rpackage version 1.6.0. Available at https://CRAN.R-project.org/package=emmeans

**Lüdecke, D** (2021) sjPlot: Data Visualization for Statistics in Social Science. Rpackage version 2.8.7. Available at [https://CRAN.R-project.org/package=sjPlot](https://cran.r-project.org/package=sjPlot)

**Macdonald P and with contributions from Du J** (2018) mixdist: Finite Mixture Distribution Moels. Rpackage version 0.5-5. Available at https://CRAN.R-project.org/package=mixdist\

**Wickham H** (2016) ggplot2: Elegant Graphics for Data Analysis. *Springer-Verlag*, New York, USA

**Wickham D and Seidel D** (2020) scales: Scale Functions for Visualization. Rpackage version 1.1.1. Available at https://CRAN.R-project.org/package=scales
